# Mountain or Sea?

**Published:** 1890-07-05

**Authors:** 


### The Doctor's Pulpit.

### MOUNTAIN OR SEA?

Summer has come at last, as it always does sooner or later, and the business of the annual holiday will have to be taken into consideration forthwith. We call it a business, because to the hard-worked and office-immured town man, and to his equally exhausted and care-harassed wife the summer trip to mountain or sea for a month or six weeks is almost as indispensable as the food and sleep of the remaining eleven months of the year. "Tut, tut," says Mr. Hardhead, "if change of air be so essential as the doctor says, how do the tens of thousands of Londoners manage to exist who never get a holiday at all?" My dear sir, we don't want to "exist," we want to live and to enjoy life, and to preserve our health so carefully during our business years as to be able to be thoroughly well and happy when old age comes. The modern man who is a practical philosopher does not kill himself in the vain effort to get rich and famous before he is forty. He lays his plans for doing his work solidly, honestly, and well until he is sixty or sixty-five, and for taking such honours as fame will give him with modest and easy satisfaction. He does not besiege the gate of fame or fortune—those fickle twins—with either miserable whinings or disgusted kicks. His pride, like that of Tennyson in his youth, makes him insist upon being on terms of equality, or, if need be, even of superiority to both fortune and fame. Thus thinking, our modern philosopher, who is of good constitution and has that considerable modicum of medical knowledge which every philosopher ought to have, may reasonably hope to spend the period of his life between the years of sixy-five and ninety in a bland and beautiful old age. As for dying at seventy, as the Shepherd King suggested, the answer of the modern philosopher to the doughty David is, that many things have happened since Goliath's head was cut off.

The choice between mountain and sea for a summer holiday should not be made to depend upon the toss-up of a penny; neither should it be decided by the prejudices of Mrs. Caudle or the whims of the children. Some people ought to go to the mountains; others should select the sea. There is often a radical difference between the air at the seaside and that on the mountain slopes. Non-medical readers should try to follow us intelligently as we strive to point out in simple language the difference between mountain and sea air, and the reasons why some persons should prefer one and some the other.

The air that comes in from the sea is for the most part both wholesome and invigorating. It is not so chemically "pure" as many people imagine; but the foreign substances that are mixed with it are not only, as a rule, quite harmless, but many of them are decidedly advantageous both to men and animals. A famous Yorkshire breeder of shorthorns, whose lands lie at least twenty miles inland from the Scarborough and Whitby coast, told the present writer two or three months ago that when the wind blows from the sea accompanied by a little hoar or mist there is such a marked change in the character of the grass that the appetites of the cattle improve in an extraordinary degree. Weakly animals become immediately convalescent, and stronger ones put on "condition" with a rapidity which would hardly be credited. This phenomenon the breeder has not observed merely once or twice, but constantly, during a period of at least forty years. It is plain, then, that sea air is of great value in certain cases.

Mountain air, on the other hand, is possessed of different qualities. Its principal characteristics are dryness and lightness. Probably most people are aware that our earth is surrounded by the atmosphere as by a gaseous envelope. But that envelope has only a certain thickness. The air is nowhere more than about five miles high; and just as a haystack is much less solid at the top than towards the bottom, so is the air less dense the higher we ascend from the earth. Moreover, air contains more or less of watery vapour mixed with it; a fact of which the least scientific may convince himself by standing on the top of a hill on a cool summer evening, and tracing out the course of a stream in the valley by means of the whitish mist which rises all along its track. It may be seen that the *visible* vapour does not rise higher than twenty or thirty feet. That is to say, as the mist ascends it becomes less and less dense, until finally it is so rare that it cannot be seen. The stream, with its vapour, well illustrates what is happening, but in a less pronounced degree, all over the surface of the earth. Water, in the form of mist, is constantly rising, though it cannot always be seen. What is certain is, that along the valleys and low grounds the air is much more moist as well as more dense than it is on the hills and mountain slopes. In fact, as is well known, the density of the air steadily diminishes with every foot of altitude we rise above the level of the sea. The higher air, as we have explained, becomes less charged with watery vapour, and, therefore, it is both drier and lighter than the lower, but it is also lighter for the same reason that the hay of the haystack is lighter towards the top; that is, because there is less air above it to press it down and make it more dense. When it is remembered that air is one of the most elastic and compressible substances known, these facts become at once both clearly intelligible and perfectly obvious.

To the seaside may safely go almost all children, most young men and women, and all those middle-aged people whose lungs are good, whose hearts are strong, who know nothing of bronchitis or asthma. Children almost invariably do well at the coast. They find an infinity of amusement on the sands; their blood is purified by the wholesome and medicated air; their appetites are invigorated; and their sleep is both sound and refreshing. Young men and women probably find the sea more strengthening as well as more enjoyable than the mountains. Boating and fishing are so pleasant and so comparatively economical that almost the whole of the day is spent in the open air. and that without tediousness or fatigue. The vast majority of middle-aged and old people may also go to the sea with pleasure and advantage. The air makes them eat, the variety of old and young life keeps them amused, the level promenade gives them easy exercise, and an occasional excursion supplies the zest of change.

"But," says the critic, "if all these classes flock to the sea who will go to the mountains?" The people who are too conscious of their lungs and hearts should seek the hills. Dry air is generally easier to breathe than moist, and a light rarefied atmosphere requires less heart power than a heavy one. Many a man who cannot breathe at all at the level of the sea breathes with comparative freedom half way up a Scotch or a Swiss mountain. Inexperienced persons make the mistake of supposing that if they were to try to live on a mountain side they would never get any exercise out of doors without climbing. That is nonsense of course. There are plenty of level and undulating plateaus two or three thousand feet up the mountain side on which months may be spent, and exercise easily obtained every day without any climbing at all. The recent epidemic of influenza has left a good many people both breathless and weak. For such the dry and rarefied atmosphere of mountainous districts is particularly suitable. Sufferers from chronic heart feebleness usually find very great comfort from living at a considerable elevation. The air being highly charged with pure oxygen satisfies the demands of the blood much more fully at each stroke of the heart than does a less highly oxydised atmosphere. The strain upon the heart is therefore greatly diminished, and opportunity is given for an accumulation of much needed cardiac energy.

All these statements may easily be proved by personal experience. The aged and the debilitated should study such elementary facts as we have set before them as carefully as they study the time-table when going on a journey, or the menu card when their digestion is in rebellion. Whatever makes life difficult tends to make it brief; and on the other hand, whatever conserves the energy prolongs the days. The month's holiday in the summer, if properly spent, should result in a health store which will not be quite exhausted before the next summer comes round. But if a mistake is made in the choice of a locality the whole holiday may be wasted, and the hard-driven man may have to return to his eleven months of duty weaker, more irritable, and more unfit for his work than when he left home. The life-contest undoubtedly becomes daily keener, and only those need expect to take first places who spend their energy with skill and recuperate it periodically with success.

